# What is the role of the neutrophil extracellular traps in the cardiovascular disease burden associated with hemodialysis bioincompatibility?

**DOI:** 10.3389/fmed.2023.1268748

**Published:** 2023-11-15

**Authors:** Jean-Paul Cristol, Alain R. Thierry, Anne-Sophie Bargnoux, Marion Morena-Carrere, Bernard Canaud

**Affiliations:** ^1^PhyMedExp, University of Montpellier, INSERM, CNRS, Department of Biochemistry and Hormonology, University Hospital Center of Montpellier, Montpellier, France; ^2^Charles Mion Foundation, AIDER-Santé, Montpellier, France; ^3^Research Institute of Cancerology of Montpellier, INSERM, IRCM, ICM, University of Montpellier, Montpellier, France; ^4^School of Medicine, University of Montpellier, Montpellier, France; ^5^MTX Consulting Int., Montpellier, France

**Keywords:** neutrophil extracellular traps, ROS, NADPH oxidase, myeloperoxidase, circulating DNA, bioincompatibility, hemodialysis, CKD

## Abstract

Despite significant progress in dialysis modalities, intermittent renal replacement therapy remains an “unphysiological” treatment that imperfectly corrects uremic disorders and may lead to low-grade chronic inflammation, neutrophil activation, and oxidative stress due to repetitive blood/membrane interactions contributing to the “remaining uremic syndrome” and cardiovascular disease burden of hemodialysis patients. Understanding dialysis bioincompatibility pathways still remains a clinical and biochemical challenge. Indeed, surrogate biomarkers of inflammation including C-reactive protein could not discriminate between all components involved in these complex pathways. A few examples may serve to illustrate the case. Cytokine release during dialysis sessions may be underestimated due to their removal using high-flux dialysis or hemodiafiltration modalities. Complement activation is recognized as a key event of bioincompatibility. However, it appears as an early and transient event with anaphylatoxin level normalization at the end of the dialysis session. Complement activation is generally assumed to trigger leukocyte stimulation leading to proinflammatory mediators’ secretion and oxidative burst. In addition to being part of the innate immune response involved in eliminating physically and enzymatically microbes, the formation of Neutrophil Extracellular Traps (NETs), known as NETosis, has been recently identified as a major harmful component in a wide range of pathologies associated with inflammatory processes. NETs result from the neutrophil degranulation induced by reactive oxygen species overproduction via NADPH oxidase and consist of modified chromatin decorated with serine proteases, elastase, bactericidal proteins, and myeloperoxidase (MPO) that produces hypochlorite anion. Currently, NETosis remains poorly investigated as a sensitive and integrated marker of bioincompatibility in dialysis. Only scarce data could be found in the literature. Oxidative burst and NADPH oxidase activation are well-known events in the bioincompatibility phenomenon. NET byproducts such as elastase, MPO, and circulating DNA have been reported to be increased in dialysis patients more specifically during dialysis sessions, and were identified as predictors of poor outcomes. As NETs and MPO could be taken up by endothelium, NETs could be considered as a vascular memory of intermittent bioincompatibility phenomenon. In this working hypothesis article, we summarized the puzzle pieces showing the involvement of NET formation during hemodialysis and postulated that NETosis may act as a disease modifier and may contribute to the comorbid burden associated with dialysis bioincompatibility.

## 1. Introduction

The development of maintenance hemodialysis (HD) for end-stage kidney disease patients is a successful therapeutic story supporting the lives of almost 3 million patients worldwide (1). Despite improvement in dialysis methods, intermittent renal replacement therapy remains an unphysiological treatment that only partially clears uremic toxins, imperfectly corrects fluid imbalance, and cyclically induces osmotic and electrolytic shifts contributing to dialysis-induced systemic stress (2, 3). In addition, repetitive blood contact with artificial material and dialysis fluid is recognized as a cause of low-grade chronic inflammation, neutrophil activation, and oxidative stress (4). Neutrophil oxidative burst can result in the extrusion of neutrophil extracellular traps (NETs), which consist of extracellular chromatin containing various granular proteins, proteolytic enzymes, and reactive oxygen species (ROS)-producing proteins. Hemoincompatibility of the extracorporeal circuit contributes to endothelium dysfunction, increases thrombogenicity, and ultimately cardiovascular disease burden (5, 6).

In recent years, the management of HD patients has evolved in two directions. First, improvements in dialysis techniques, such as the use of ultrapure dialysate, online hemodiafiltration (HDF), and surfaces with reduced complement activation, have significantly enhanced biocompatibility. Conversely, HD patient demographics have shifted from younger individuals with minimal comorbidities to older, frail patients with preexisting complex comorbidities. The intricate interplay between neutrophil activation, platelets, and endothelial cells plays a crucial role in HD-induced endothelial dysfunction, potentially exacerbating vascular impairment and organ damage in this vulnerable population.

In this working hypothesis article, we provide a concise overview of the contribution of HD bioincompatibility to dialysis-induced systemic morbidity. We postulate that dialysis triggers an innate immune response through the formation of NETs. Given that uncontrolled NET formation or NETosis has previously been identified in various inflammatory systemic diseases, NETs could be a pivotal piece in the bioincompatibility puzzle, potentially contributing to the development of vascular diseases and poor outcomes in HD-treated patients.

## 2. Imbalance and maladaptive response to NET formation contribute to poor outcomes of major systemic inflammatory diseases in the general population

### 2.1. NET as a first-line defense against microorganisms

**Figure 1:**
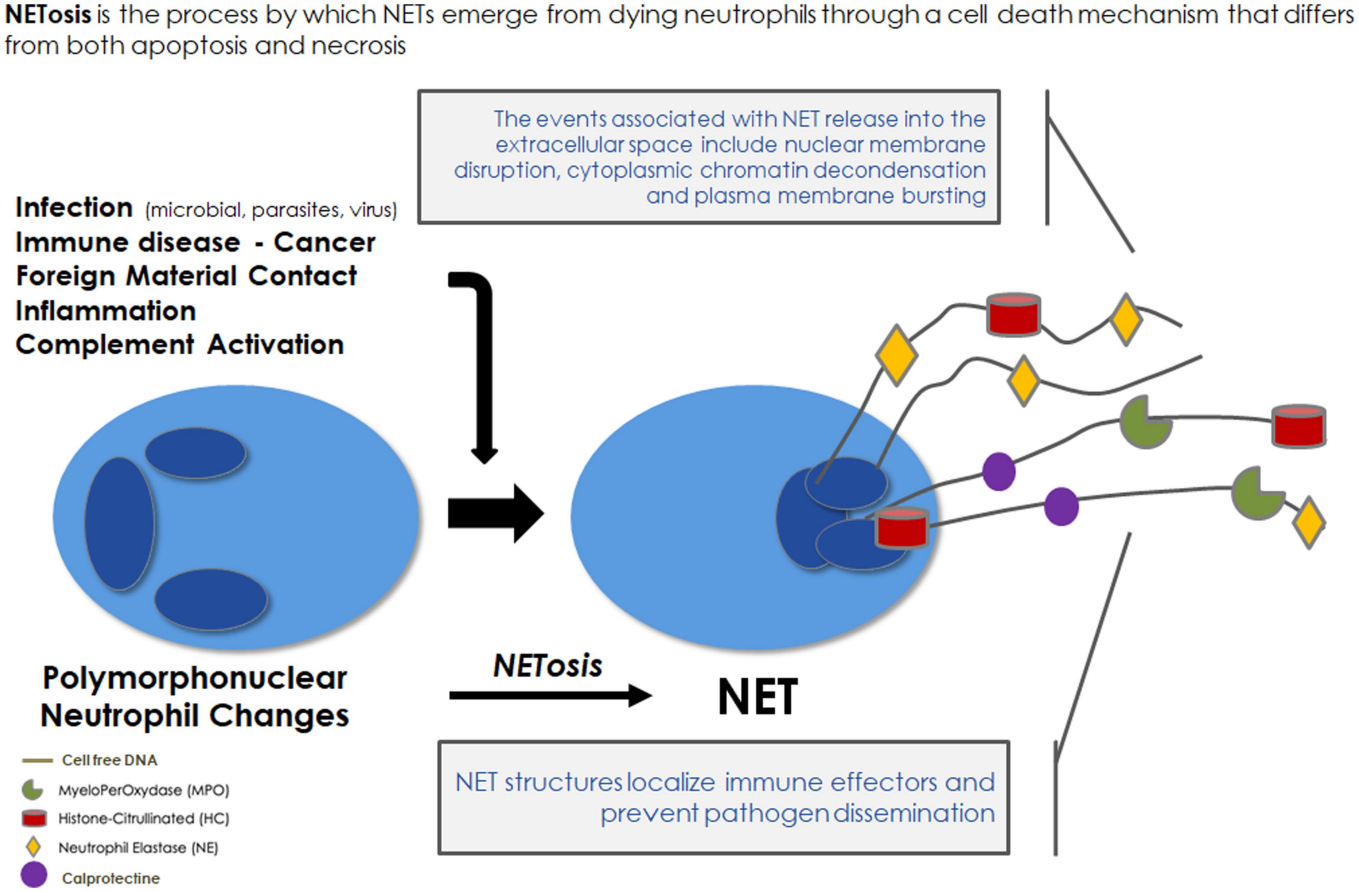
Neutrophil extracellular traps: formation and role in immune defense. NET, neutrophil extracellular traps.

Neutrophils are regarded as the first line of innate immune defense. A part of neutrophil function aims at producing NETs, which were discovered only two decades ago (7) and were first described as a special form of programmed cell death different from necrosis and apoptosis (8). In response to stimuli, NETs extracellularly release extensive fibrous structures mainly composed of chromatin containing granulomatous and cytosolic proteins assembled on a scaffold of released chromatin. The classical NETosis is strictly dependent on NADPH oxidase activation (9). ROS overproduction induces neutrophil degranulation leading to the release of the protein complex “azurosome,” which includes serine proteases, such as neutrophilic elastase (NE), cathepsin G, azurocidin, and S100 family proteins, such as calprotectin (10) and myeloperoxidase (MPO) (Figure 1). Serine proteases as well as MPO could break down lamin and histones contributing to the chromatin decondensation and destruction of the nuclear envelope. At the final stage of NETosis, pores are formed in the plasma membrane and chromatin is released into the environment with NET formation. Proteins released from the granules are strongly bound to the decondensed chromatin (9). These structures physically entrap microbes and exploit the strong enzymatic power of anchored enzymes and coagulant function to suppress their dissemination. In addition, NET byproducts (nuclear and mitochondrial DNA fragments associated with nucleosomes, histones, granule proteins, etc.) trigger an inflammatory response. Thus, NETs appear to have a “double-edged sword” function. On the one hand, NETs participate in an early efficient response to neutralize microorganisms. On the other hand, NET byproducts in exaggerated or uncontrolled amounts may be harmful to the host by being toxic to endothelial cells and parenchymal tissue (11) and by leading to immunothrombosis (12).

### 2.2. NET-associated toxicity

NETs are macromolecular complexes, and their byproducts in excess may potentially lead to toxic effects. As they are the major NET constituents, nucleic acids appear as the most toxic ones mainly due to their high degradation in the blood resulting in the accumulation of circulating DNA (cirDNA) in the form of mononucleosomes and circulating mitochondrial (mt) DNA. The presence of cirDNA was first described in 1977, and chronic vascular toxicity was hypothesized (13). Both types of nucleic acids appear to be the primary source of damage-associated molecular patterns (DAMPs), also known as alarmins. These endogenous molecules are recognized by pattern recognition receptors (PRRs), and their activation leads to the synthesis of proinflammatory cytokines, inducing inflammation (12). Additionally, short DNA fragments from mononucleosomes are recognized by DNA sensors such as Toll-like receptor 9 (TLR9), which is more specific for microbial DNA, and stimulator of interferon genes (STING), capable of recognizing a wide range of DNA. TLR9 and STING are located in endosomes and the cytosol, respectively. This recognition triggers the secretion of type I interferons and proinflammatory cytokines, thereby regulating host defense mechanisms. Histones from mononucleosomes were reported to be toxic by triggering inflammation as well. Because of its bacterial antecedent, mtDNA can serve as a DAMP, in particular, mtDNA can bind to the TLR9 receptor of leukocytes via hypomethylated CpG motifs and trigger an inflammatory reaction (14). As exacerbated inflammation has been associated with a failure in innate immune tolerance to self-DNA, in particular in inflammatory disorders, it is hypothesized that cirDNA, mostly of mitochondrial origin, may be a mediator of transfusion-related acute lung injury (15). It was shown that mtDNA DAMP levels may predict the development of acute respiratory distress syndrome after multiple transfusions (16).

In addition to DNA and histones, granule proteins such as MPO and NE also contribute to the triggering of an inflammatory process. In the presence of hydrogen peroxide and halides, MPO catalyzes the production of hypochlorous acid, the latter could generate long-lived oxidants, such as chloramines, enhancing long-lasting oxidative stress (17). Multiple lines of evidence demonstrate that MPO is associated with oxidative stress and inflammation (18, 19). MPO activation is involved in endothelial dysfunction, reduced NO availability, and impaired vasoreactivity leading to cardiovascular disease (18). During inflammatory cardiovascular disease, MPO could be released by activated leukocytes and transported to the endothelium resulting in sequestration and accumulation within the vascular endothelium. Thus, the synergistic action of NADPH oxidase and MPO activation could be a pivotal event in NET-induced endothelial dysfunction and vascular damage (20). In addition, MPO-damaged biomolecules can act as chemo-attractants and modulate gene expression enhancing inflammation and tissue damage (19, 21). NE is known as one of the most destructive enzymes in the body and overwhelming NE release together with simultaneously produced ROS can cause local tissue injury and neutrophil-mediated endothelial injury^1^ (22).

### 2.3. NETs as components of inflammatory diseases

Unbalanced NET formation and maladaptive response were observed in infectious and non-infectious diseases (11, 12, 23). They have been observed in inflammatory diseases, such as pre-eclampsia, small-vessel vasculitis, and systemic lupus erythematosus (SLE). The deleterious role of NETs has been particularly studied in the context of autoimmune diseases, especially lupus. NETs expose many autoantigens in the extracellular space, in particular double-strand DNA or MPO. Numerous studies have demonstrated the existence of the deregulation of NETs during lupus. Increased circulating levels of NETs and evidence for NET in SLE patients’ tissue are associated with the number of immune complexes typically containing nucleic acids associated with various proteins, such as the chromatin-associated protein HMGB1, the antimicrobial peptide LL39, ribonucleoproteins, and others (24). Of note, Elkon (25) found in patients with SLE an impaired ability to degrade NET. The unbalanced NET formation was also shown in acute respiratory distress syndrome, acute lung injury, diabetes, and sepsis and was suggested in sickle cell disease, cystic fibrosis, periodontitis, autoimmune vasculitis, rheumatoid arthritis, severe obesity, pre-eclampsia, or kidney diseases (12).

NET deleterious effects were observed in infectious diseases as well. Microbe-induced respiratory illnesses such as lung endothelial injury were associated with high NET formation. Among the numerous studies reporting it, NETs were detected in the bronchoalveolar lavage fluid of children with severe respiratory syncytial virus infection of the lower respiratory tract (26). In addition, pathologies of chikungunya virus, simian immunodeficiency virus, influenza, parvovirus, rhinovirus, and influenza pneumonia involve neutrophils and NETs (12). Among infectious respiratory diseases, NETs have been involved in SARS-COV-2 infection (27, 28). Recently, it has been shown that NETosis was a key factor for acute respiratory distress (12, 27). Interestingly, NETosis persists in the post-acute COVID phase, suggesting a feedback loop to NET formation due to the combined stimulation (i) from the NETs byproducts, in particular, the cirDNA fragments acting as DAMPs and neutrophil activation; (ii) from the subsequent platelets activation; and (iii) the generation of auto-antibodies (29). In this context, the COVID post-acute phase NETosis could enhance the uremia- and HD-induced low-grade inflammation and could account in part for the higher risk of death in patients on maintenance HD (30, 31).

In brief, the unbalanced formation of NETs contributes to vascular disease progression in various diseases associated with chronic inflammatory processes (12, 32). NETs result from neutrophil degranulation induced by ROS overproduction via NADPH oxidase, consisting of a mixture of modified chromatin, cirDNA, and bactericidal proteins (Figure 1). Despite its role in the vascular disease pathway, NETosis remains poorly investigated in HD. As NET components could be taken up and could lead to damage in the endothelium, these components might be considered as a marker of vascular memory of intermittent bioincompatibility phenomenon.

## 3. NET formation and link with bioincompatibility of the extracorporeal circuit: highlighting the prominent role of the hemodialyzer acting as bioincubator

### 3.1. Bioincompatibility phenomenon

Bioincompatibility (or more specifically, hemobioincompatibility) of the extracorporeal circuit is recognized as a cause of dialysis-related low-grade inflammation contributing to morbidity and mortality through systemic effects (33). HD allows the removal by diffusion and convection of low molecular weight uremic toxins and excess water by the use of a semipermeable polymeric membrane in an extracorporeal system (2, 34). However, intermittent HD remains an imperfect treatment that only partially corrects uremic abnormalities and could be harmful to patients. The “unphysiological” nature of intermittent HD and HD-induced systematic stress have long been recognized as a leading cause of dialysis intolerance (35). Bioincompatibility complex syndrome results from the interaction of blood with multiple components of the extracorporeal circuit. In brief, as soon as blood leaves the vascular component and enters the extracorporeal circuit, it encounters multiple stimuli that contribute to the activation of a cascade of serum proteins and blood cells. Dialysate itself is a potential biological stressor. Indeed, the chemical composition including acetate or citrate could result in metabolic stress (36). On the other hand, dialysis fluid contaminants, bacterial translocation, and bacterial-derived products (lipopolysaccharide and muramyl dipeptide) could activate leukocytes (2). Blood–dialyzer interaction that occurs in the warm aquatic environment of the dialyzer creates the perfect conditions for a bioincubator to nurture biocell reactions. Other exogenous stressors including venipuncture, blood–air interface, mechanical cell trauma (i.e., pump), nature of blood tubing, and contact with foreign material of the extracorporeal circuit contribute to acute phase reactions (37). Artificial membrane packed in the hemodialyzer represents the centerpiece of HD, and the material of the dialysis membrane bathed by dialysis fluid and its components are the main determinants of hemobioincompatibility. HD strategy has developed membrane technology in order to better clear uremic toxins and improve biocompatibility. Indeed, the use of synthetic membranes [made of polysulfone, polyethersulfone, poly(methyl methacrylate), polyester polymer alloy, polyacrylonitrile, polycarbonate, and polyamide] has allowed an improvement of patient outcomes compared to the first HD cuprophan membranes based on cellulose derived from cotton. New strategies have been considered in the development of HD membranes by incorporating bioactive compounds, such as vitamin E (38), lipoic acid, or heparin (39). Recently, a new generation of dialyzer membranes emerged, comprising a blend of polysulfone and polyvinylpyrrolidone (PVP), with small amounts of α-tocopherol added to stabilize the blood-side surface of the membrane. The robust hydrophilic layer on the membrane surface enhances the hemocompatibility of polysulfone membranes through improved antifouling ability, which yields less protein adsorption and lower coagulation activation (40).

### 3.2. Dialysis-related inflammation, enhanced role of dialysis fluid contamination

Observational studies have shown that low-grade inflammation, as assessed by highly sensitive C-reactive protein (CRP), was highly predictive of cardiovascular disease (CVD) in the general population (41, 42), advanced chronic kidney disease patients (43) and dialysis-dependent patients (44). The CRP increase results from an imbalance between pro-and anti-inflammatory cytokines involving several factors. The first hit is the decrease in glomerular filtration rate (GFR), which is associated with an increase in pro-inflammatory cytokines (45). Further hits contribute to the low-grade inflammation being part of the uremic complex syndrome, such as toxin accumulation (46), vascular access type (47), intercurrent or subclinical infection including periodontal disease (48), cytokine genetic background, or dialysis-induced mainly mediated through bioincompatibility reactions. As a result, the net prominence of proinflammatory cytokines contributes significantly to the poor outcome of end-stage kidney disease patients (33, 49).

Although high-sensitive CRP and interleukin levels have been extensively reported as biomarkers of uremic-and dialysis-induced inflammatory response, they have serious limitations when exploring bioincompatibility pathways. With the improvement of dialysis modalities (i.e., high-flux synthetic polymer with better biocompatible profile) and generalization of ultrapure dialysis fluid, CRP levels have significantly decreased in dialysis patients. As an example, the median CRP locally reported by our group reached 7.5 mg/L at the beginning of the century (44), further decreased at 5.7 mg/L in the FRENCHIE randomized clinical trial study (50) down to 4 mg/L in the Fragil HD study (unpublished data). However, low-grade inflammation persists in a geriatric population from the South of France with a median CRP value of 2 mg/L (42). In addition, cytokine dosages are more difficult to accurately assess in a clinical setting. Indeed, dialysis technique, high-flux membranes used in HD or HDF alone have no significant impact on predialysis plasma cytokine levels (50), indicating that membrane type or fluxes are not sufficient *per se*, but highlighting the necessity of using ultrapure dialysis fluid to achieve such reduction. Provided these conditions are fulfilled, predialysis levels of proinflammatory cytokines and CRP might remain stable for 2 years, which could rule out any cumulative effect of intermittent stimulation (50). Finally, plasma cytokine levels in dialysis patients reflect a delicate balance between cytokine production from the patient side and cytokine removal from the dialysis side. As a consequence, cytokine level changes observed during dialysis sessions could not be considered sensitive biomarkers of bioincompatibility.

### 3.3. Biological pathways of dialyzer bioincompatibility

In order to explore more precisely the bioincompatibility phenomena and pathways, we have to expand the scope of our canonical markers to understand which ones are the components of bioincompatibility reactions. Although several dialysis-related factors such as exposure to endotoxin from dialysis fluid, silent vascular access infection, and dialysate chemical composition could be involved in bioincompatibility reactions, blood interaction with the dialyzer membrane is usually assumed to be the main component. Contact phase activation, complement pathway, leukocyte activation, and thrombogenicity are well-identified pathways of bioincompatibility. Platelet binding to the membrane is enhanced by protein adsorption such as collagen, fibronectin, and fibrinogen, which could activate in turn glycoprotein IIb/IIIa receptors. Activation of platelets may then in turn initiate the clotting cascade leading to the thrombus formation (51, 52). Complement activation is also well recognized as a key event of bioincompatibility, in particular with cellulosic-based membranes. However, complement activation is an early and transient event that tends to turn down across dialysis sessions leading to the normalization of anaphylatoxin levels at the end of the dialysis (52, 53). This transient complement activation has been shown to trigger leukocytes and other immunocompetent cells leading to proinflammatory mediator secretion with a subsequent oxidative burst and decrease in antioxidant defense and oxidative stress (54).

### 3.4. NADPH oxidase and MPO activation as main triggers of dialyzer bioincompatibility

Once activated, immunocompetent cells are able to produce large amounts of cytokines and ROS which are clearly linked by amplification loops (55, 56). Among the functional consequences of neutrophil activation, the enhancement of ROS production results in the translocation and the assembly of cytosolic components of the NADPH oxidase enzyme (57). The activated NADPH oxidase complex catalyzes the reduction in molecular oxygen O_2_ into superoxide anion O_2_^−^, which then rapidly dismutates to form hydrogen peroxide, a reaction catalyzed by superoxide dismutase. Thereafter, hypochlorous acid and chloramines could be produced by MPO (17). In addition, O_2_^−^ could react with large amounts of NO° produced by the inducible NO synthase, leading to a deleterious peroxynitrite (ONOO^−^). Such reactions triggered by the NADPH oxidase are beneficial for bacterial killing in healthy people but represent an additional deleterious source of ROS production in chronic kidney disease patients.

NADPH oxidase activation and cytokine secretion are closely linked. As such, proinflammatory cytokines induce the activation of NADPH oxidase. ROS overproduction may stimulate proinflammatory transcriptional factors such as NFκB, which in turn may increase the synthesis of cytokines and may further activate the NADPH complex.

MPO activity is increased in chronic kidney disease patients and further enhanced in HD according to the type of membrane and contamination of dialysis fluid. Recently, it has been shown that, despite using biocompatible membranes including polysulfone, vitamin E-bonded type, polyester type, or PMMA, MPO was significantly increased after dialysis and was associated with polynuclear cell apoptosis (58). In addition to its direct effect on the endothelium, MPO activation leads to protein carbamylation (59–61). These non-enzymatic post-translational-derived product modifications alter the physiologic functions of proteins and could promote new deleterious properties. Carbamylation affects circulating proteins, such as hemoglobin, albumin, and low- and high-density lipoproteins. Carbamylation modification has also been evidenced in tissue proteins, such as collagen, elastin, and mitochondrial proteins (62). Such protein modifications enhance vascular disease progression and the occurrence of metabolic complications, such as erythropoietin resistance, hemostatic dysfunctions, immune response disorders, insulin resistance, vascular damage, and ultimately poor outcome (62). Interestingly, endothelial and vascular smooth muscle cells do not express the MPO enzyme. However, after leukocyte activation, MPO could be released and bind to endothelial cells and the extracellular matrix of vascular cells (63). Since after exposure to urea, ROS overproduction is sustained in endothelial cells for up to 48 h, one can postulate that colocalization and coactivation of NADPH oxidase and MPO have a remaining, long-lasting vascular effect after dialysis session during the interdialytic period (64). MPO has been identified as a predictive biomarker of poor outcomes in patients with chest pain reflecting coronary ischemic insult and in HD patients (65, 66).

### 3.5. NETosis as a unifying process of bioincompatibility: putting the pieces of the puzzle together

**Figure 2:**
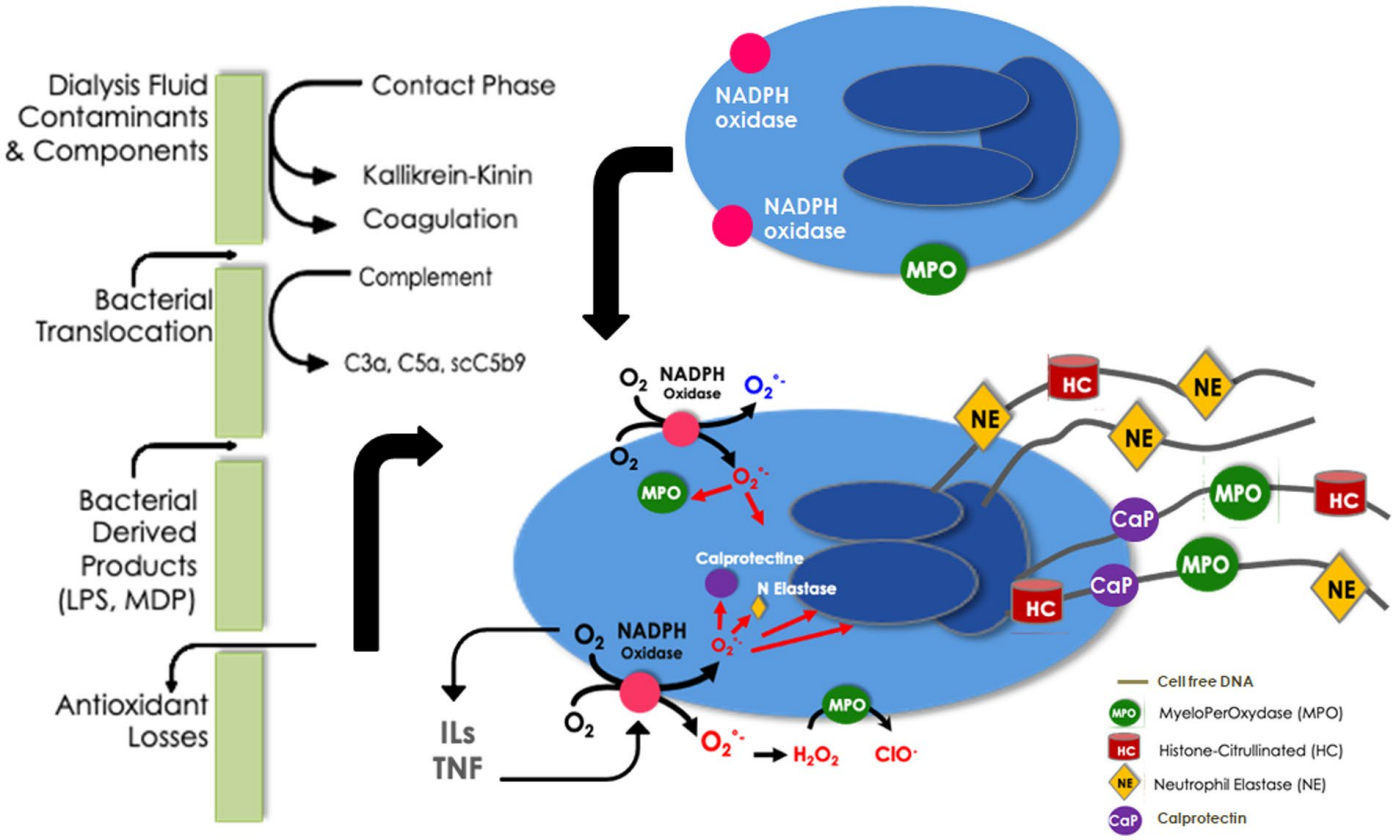
Neutrophil extracellular traps in bioincompatibility: formation and factors implicated in dialysis-induced systemic stress.

NETosis has been evidenced during HD mainly by exploring cirDNA and its involvement in bioincompatibility phenomenon has been suggested (67). However, there is no “gold standard” for detecting NETs in blood or in tissue and NETosis may be easily evidenced by the detection of surrogate biomarkers, such as cirDNA, MPO, or elastase. Several pieces of the NETosis puzzle have been identified in HD (Figure 2). ROS overproduction and NADPH oxidase stimulation acting as starting points of NETosis have been extensively reported in this setting. In addition, MPO release, one of the main protein components of NETs, has been evidenced during HD sessions. Furthermore, the serine protease elastase, another component of NETs, which is significantly increased in end-stage kidney disease patients, is further enhanced after 1 h of HD (68). Exacerbated elastase activity in uremic patients prior to dialysis sessions has been reported by Khatib-Massalha and colleagues (69) and elastase concentration increases progressively during dialysis sessions (70). By contrast to the early and transient activation of complement, elastase activity is enhanced after 1 h of dialysis and then sustained till the end of the session (40). Using three different synthetic polymer membranes, the new polysulfone-based FX CorAL 600 (Fresenius Medical Care, Bad Homburg, Germany), the polyarylethersulfone-based Polyflux 170H (Baxter Healthcare Corporation, Deerfield, IL), and the cellulose triacetate-based SureFlux 17UX (Nipro Medical Europe, Mechelen, Belgium), it has been shown that activation of elastase was dependent on the membrane nature and maybe on other components such as sterilization process (40). Furthermore, as recently shown, the surface modification of the new polysulfone membrane, the so-called hydrophilic layer, resulting from a PVP enrichment and a stabilization by alpha-tocopherol of the polysulfone polymer, decreases significantly complement activation and elastase release. It is noteworthy that early complement activation is significantly reduced but not entirely eliminated with the use of new membranes and features. As complement activation could trigger NETosis, it is important to consider this residual complement activation when planning for future complementary actions (40).

NETosis has been recently evidenced during a dialysis session using a multimarker approach, through a simultaneous increase in cirDNA and a release of MPO and calprotectin (71). Moreover, it has been shown that endothelial dysfunction, assessed by the kinetic of adhesion molecules, was also related to NET occurrence (71). *In vitro*, uremic serum obtained from HD was shown to increase NET formation, MPO, and nucleosome release from polymorphonuclears and induce apoptosis of endothelial cells (72). Also, neutrophils isolated from HD patients tend to express an increased basal level of ROS production and cirDNA release, suggesting a uremia-induced NET formation (73). However, after PMA or lipopolysaccharide stimulation, oxidative burst and NETosis are significantly decreased in agreement with the decreased capacity of HD neutrophils to kill extracellular bacteria and an increased susceptibility to infections in these patients (74).

Levels of cirDNA were positively correlated with elastase concentrations, inflammatory markers, and oxidative stress as assessed by the lipid peroxidation index (75). In this context, recent outcome-based studies have shown that cirDNAs were the best predictors of all-cause and cardiovascular mortality in end-stage kidney disease patients at 1 year, in both unadjusted and adjusted models for basic confounding factors (75, 76). Interestingly, it has been shown in previous studies that cirDNA increase was triggered by HD sessions (77). Furthermore, in a multivariate model exploring the role of cirDNA kinetic (pre- and post-dialysis) on mortality, it has been reported that only post-dialysis cirDNA levels were predictors of poor outcome (78). It is therefore tempting to postulate that cirDNA released during dialysis sessions reflects such NET formation which then contributes to patient outcome worsening.

The direct link of cirDNA with NETs as well as considering cirDNA as a NET marker may be based on the recent study demonstrating that NETs lead to the production of circulating mononucleosomes, which were shown as the main components of cirDNA-associated structures. The detection of cirDNA in blood is typically associated with the protective effect of mononucleosomes, which determines its fragmentation (79). The concomitant observation that (i) mononucleosomes clearly accumulate in cirDNA extracts after incubation of very high molecular weight DNA in serum or plasma extracts of cirDNA from mice or human subjects, in combination with (ii) the observation that activated neutrophils in culture produce NETs and high molecular weight DNA, which are further degraded mainly into mononucleosomes, demonstrates that, in a physiological setting, NETs can produce cirDNA as a byproduct, independent of apoptosis. These observations directly and unequivocally provide the mechanistic bridge linking NETs and cirDNA, advocating the use of cirDNA fragments or mononucleosome amounts as NET markers.

## 4. NETosis as a significant component of dialysis-induced cardiovascular disease burden: an attractive working hypothesis for improving biocompatibility

**Figure 3:**
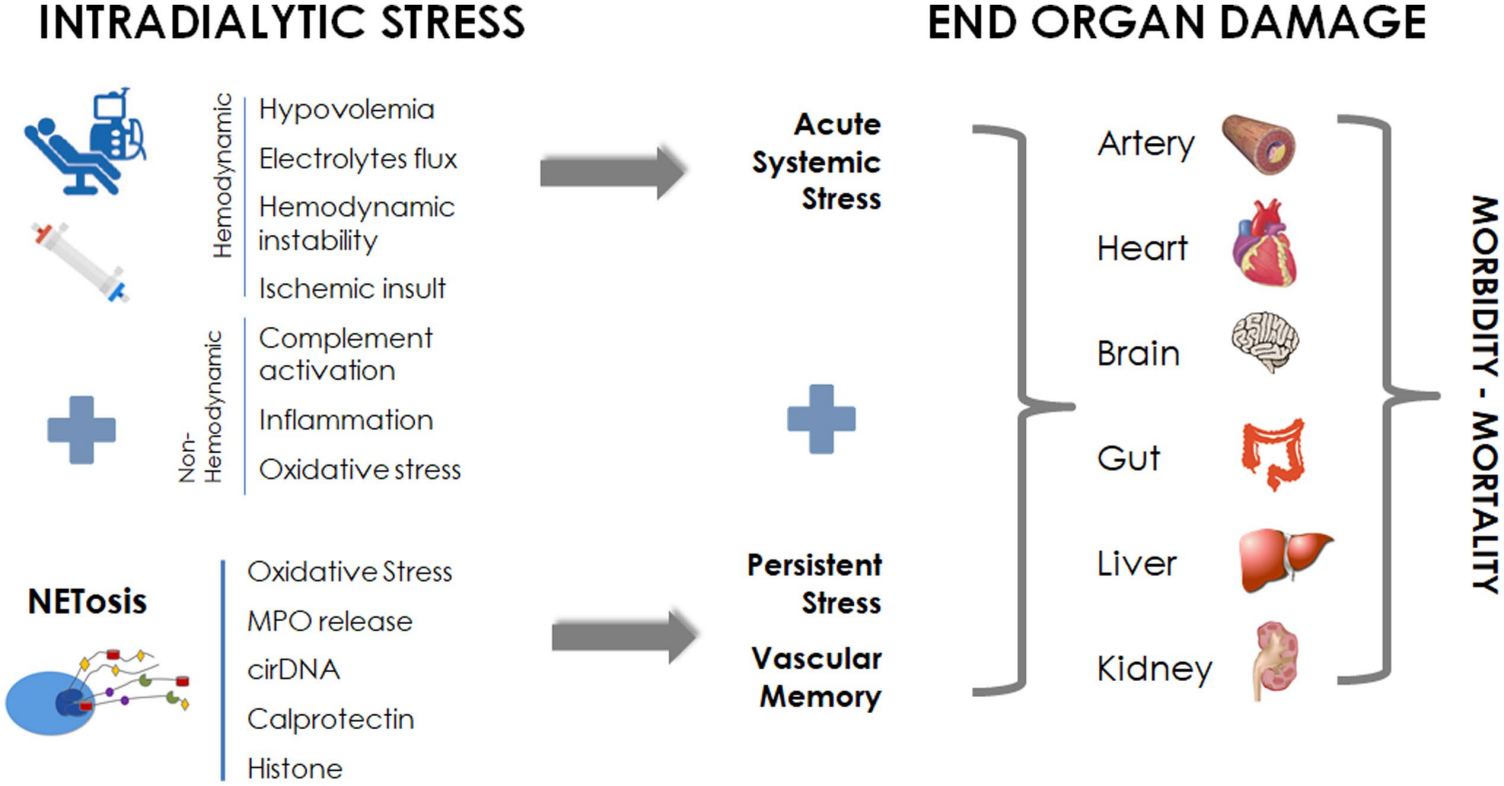
NETosis in dialysis: contribution to dialysis-induced systemic morbidity.

Hemodialysis patients are exposed to periodic “systemic circulatory stress” resulting from the treatment schedule, leading to a multiorgan injury superimposed on preexisting comorbidities (2, 3, 80). Intermittent HD (e.g., conventional thrice-weekly 4-h treatment schedule) generates cyclical fluctuations in volume (hyper to hypovolemia), blood pressure changes, osmotic shifts and swings in solutes, and electrolyte levels (35). Dialysis-induced systemic stress resulting from the “unphysiology” of intermittent dialysis is dramatically worsened by bioincompatibility phenomena that conspire with the limited efficiency of the overall renal replacement therapy. In addition, conventional high-flux HD has limited effect on the removal of medium- and high-molecular-weight uremic compounds as well as on protein-bound uremic toxins, suggesting a “residual uremic syndrome” contributing to patient morbidity (3). All these factors contribute to patients’ end-organ damage, including vasculature, cardiac, gut, liver, and cognitive dysfunction, and negatively impact patients’ perception and quality of life (Figure 3).

The endothelial system has been identified as a critical target pathway in dialysis-induced multiorgan morbidity. NETosis emerges as a key player in the interplay between endothelial cells, platelets, and neutrophils. It has been demonstrated that adhering cells onto the dialyzer membrane at the end of a dialysis session primarily activate neutrophils, with the presence of NETs detectable via immunofluorescence (81). Interestingly, NETs are associated with thrombin generation in a tissue factor (TF)-dependent manner. Furthermore, the presence of TF mRNA in adhering neutrophils has been confirmed (81). Recent research has shed light on the interaction between NETs and platelets (20), as NETs can express tissue factor, further triggering thrombin generation and platelet activation, ultimately increasing the thrombogenic potential of NETs. Conversely, activated platelets could stimulate neutrophils to form NETs (82). The NET network could impede blood flow and promote intravascular occlusion of blood vessels independently of plasmatic coagulation (83). Therefore, NET activation could significantly favor microthrombus formation and endothelial dysfunction.

Additionally, both NETs and extracellular vesicles (EVs) are simultaneously released upon activation. However, it remains unclear whether NETs and EVs act as antagonistic, synergistic, or independent mediators (84). Nevertheless, EV-NET complexes can also form and seem to synergistically enhance the procoagulative and prothrombotic capacity of NETs by delivering tissue factor (85). EV contains lipids, informative molecules, and coding or non-coding mRNA. Microvesicles could thus induce phenotypic changes in endothelial cells (86, 87). It has been reported that procoagulant microvesicles are increased in HD patients. Therefore, NETs and microvesicles may conspire to induce a procoagulant milieu in HD patients (88, 89).

NET formation occurring during a single HD session might be related to acute-phase inflammation. However, the repetition of dialysis sessions almost every other day might result in chronic low-grade inflammation, making it challenging to assess the contribution of NETosis to this biological systemic stress. Interestingly, NETosis, such as elastase or cirDNA release, appears as a late event in the bioincompatibility phenomenon, rising after the first hour of dialysis and persisting until the end of the session, raising questions about sustained NETs during the interdialytic period (40, 78).

During the interdialytic period, several factors affecting the kinetics and circulating levels of NETs must be considered. First, an increase in uremic toxins could activate polymorphonuclears, resulting in NET formation. Indeed, it has been shown that uremic toxins induce DNA–histone complexes in neutrophils *in vitro* (90). Additionally, cirDNA has been proposed as a potential biomarker of renal damage (91). MPO and elastase levels have been reported to be elevated in chronic kidney diseases prior to dialysis (69, 92, 93) but they are enhanced in dialysis patients compared with end-stage renal failure patients (68). On the other hand, the disappearance rate of NETs after a dialysis session is unknown. CirDNA is rapidly degraded in the blood due to the numerous plasma DNases (67, 76). However, DNase expression or activity could be decreased in chronic pathologies, increasing the half-life of cirDNA. In contrast to cirDNA, MPO could be bound and sequestered in the vascular wall (18). In association with the prolonged urea-induced NADPH oxidase stimulation (64) activity, the binding of MPO to endothelial cells (63) could produce hypochlorous acid and chloramines after the end of the dialysis session, potentially exerting a long-lasting effect on endothelial function. The interdialytic kinetics of NET components warrant further investigation, but it is tempting to speculate about a “vascular memory” of acute bioincompatibility phenomenon linked to the potential persistence of NETs, as observed after an acute phase of viral insult (12, 29). Several recent studies tend to support this concept. Indeed, in a recent prospective study, NETosis was associated with endothelial dysfunction and positively correlated with a reduced flow-mediated dilation of the brachial artery despite adjustment for cardiovascular risk factors and uremic toxin concentrations (94). It has been also shown that NETosis was able to induce platelet trapping and promote microvascular occlusions (95, 96) leading to multiorgan failure. NET was also detected as essential for the development of profound venous thrombosis (97). The prothrombotic effect of NETs, mainly initiated by platelet activation, is further enhanced by DNA-bound elastase that degrades plasminogen, reduces plasmin formation, and decreases fibrinolysis (98). Here, again these prothrombotic actions could be sustained during the interdialytic period.

In addition to this prothrombotic effect, NETosis might be involved in CV diseases (20), a leading cause of mortality in dialysis patients. Recent intradialytic imaging studies have evidenced a reduction in myocardial perfusion and contractility (99). Dialysis-induced cardiac disorders, known as cardiac stunning, are due to transient ischemia injury resulting in myocardial death and fibrosis. HD session *per se* has been shown to be a significant hemodynamic stressor able to precipitate such recurrent ischemic insults (100). Furthermore, NETs have been identified as key contributing components in ischemia–reperfusion injury as shown in the myocardial no-reflow phenomenon (101). NET formation participates in the extension of myocardial infarction size as confirmed by large amounts of NETs in coronary thrombi (102). Surrogate markers of NET burden as cirDNA and citrullinated histones were increased in the infarct site (103). Levels of cirDNA and infarct size were found to be strong and independent predictors of in-hospital major adverse CV events (104). Such a positive association between high levels of cirDNA, nucleosomes, MPO complexes, and ST-elevation myocardial infarction with poor patient outcomes has been already described (105). In HD patients, high levels of cirDNA have been associated with major cardiovascular events (90). By contrast, a progressive decrease in these biomarkers was observed after a successful revascularization procedure (106). Finally, markers of NETs are associated with adverse clinical outcomes in stable coronary artery disease patients (107).

It has been evidenced that cerebral vascular disease of HD patients could translate into brain alteration, cognitive impairment, and morbi-mortality (108). Intradialytic Doppler ultrasonography and cerebral magnetic resonance imaging have recently established a link between the deleterious effects of HD and cognitive function as mediated by changes in cerebral arterial flow velocity (109, 110). In a prospective observational cohort study of 97 adults receiving HD, the degree of decline in flow velocity in the middle cerebral arteries correlated with the decline in cognitive function (110). Moreover, repetitive HD-induced brain ischemia has been implicated in white matter damage and leukoaraiosis development (111). Recently, it has been also shown that surrogate NET biomarkers, such as cirDNA, MPO, and calprotectin, were increased in stroke patients (112) while NETs exacerbate ischemic brain injury (113). The NET formation is also accompanied by reduced neovascularization and increased blood–brain barrier damage (114).

The gut and liver are also targets of dialysis-induced hemodynamic stress. Dialysis-induced acute circulatory stress contributes to the development of gastrointestinal dysfunction and “gut stunning” (6). As shown, gut ischemia leads to bacterial endotoxin translocation and the release of gut-derived uremic toxins from the gut to the bloodstream (115). Gut-derived uremic toxin and endotoxin levels are contributing to maintaining low-grade inflammation in HD patients (116, 117). Circulating endotoxin levels are increased in dialysis patients and correlate with intradialytic instability, systemic inflammation, cTnT levels, ultrafiltration rate, dialysis-induced myocardial stunning, and risk of subsequent mortality (115). NETs are increased in the inflamed intestinal mucosa, feces, or blood, and NET abundance positively correlates with the degree of inflammatory intestinal disease (118, 119). In an experimental intestinal ischemia–reperfusion injury model, NETs were involved in organ damage and intestine inflammation (120). Interestingly, NETs were able to damage the cytoskeleton of human enterocyte-like cells and destroy the intestinal epithelium facilitating bacterial translocation (121).

In brief, all these facts strongly suggest that dialysis-induced systemic stress and NETosis process that act synergistically during dialysis may have a long-lasting systemic effect contributing to end-organ damage and morbidity of dialysis patients (Figure 3).

## 5. Conclusion and perspectives

Bioincompatibility in HD is driven by complex molecular and cellular pathways that intricately act in a cascade of phases. To outline this process schematically, the initial event involves protein adsorption, platelet activation, and complement activation, occurring within the first 15 min of a dialysis session. This transient activation sets the stage for prolonged leukocyte activation, which persists until the end of the dialysis session. Leukocyte activation leads to a cytokine storm, accompanied by an overproduction of ROS due to NADPH oxidase activation. Subsequently, oxidative stress amplifies the production of NETs, resulting in the release of serine proteases, neutrophil elastase, MPO, calprotectin, decondensed chromatin, and cirDNA. NETosis emerges as a late event in the bioincompatibility phenomenon, and the interplay between NETs, platelets, and coagulation may persist during the interdialytic period, suggesting a “vascular memory” of dialysis-induced bioincompatibility. From this perspective, NETs can be considered as markers of the vascular memory of dialysis-induced bioincompatibility, contributing to the systemic circulatory stress observed in dialysis patients. The ongoing activity of NETs during the interdialytic period may, in turn, impact systemic circulation, contributing to end-organ damage and dialysis-induced systemic morbidity. Further studies are needed to investigate the kinetics of NETosis during both intradialytic and interdialytic periods, assessing NET involvement in target organs affected by systemic stress. In this context, it will also be interesting to explore the relative contributions of more efficient renal replacement therapies, such as intensive dialysis treatment schedules (e.g., nocturnal or daily treatments), versus more biocompatible hemodialysis, to the NET imbalance phenomenon. As DNase and anti-coagulant treatments have been proposed for NET-related diseases, these therapeutic options warrant further investigation (12).

## Author contributions

J-PC: Conceptualization, Writing – original draft. AT: Conceptualization, Writing – review & editing. A-SB: Writing – review & editing. MM-C: Writing – review & editing. BC: Writing – review & editing.

## Footnotes

^1^ https://clinicaltrials.gov/ct2/show/NCT04338126
