# Corrigendum: What is the role of the neutrophil extracellular traps in the cardiovascular disease burden associated with hemodialysis bioincompatibility?

**DOI:** 10.3389/fmed.2024.1393023

**Published:** 2024-03-12

**Authors:** Jean-Paul Cristol, Alain R. Thierry, Anne-Sophie Bargnoux, Marion Morena-Carrere, Bernard Canaud

**Affiliations:** ^1^PhyMedExp, University of Montpellier, INSERM, CNRS, Department of Biochemistry and Hormonology, University Hospital Center of Montpellier, Montpellier, France; ^2^Charles Mion Foundation, AIDER-Santé, Montpellier, France; ^3^Research Institute of Cancerology of Montpellier, INSERM, IRCM, ICM, University of Montpellier, Montpellier, France; ^4^School of Medicine, University of Montpellier, Montpellier, France; ^5^MTX Consulting Int., Montpellier, France

**Keywords:** neutrophil extracellular traps, ROS, NADPH oxidase, myeloperoxidase, circulating DNA, bioincompatibility, hemodialysis, CKD

In the published article, there was an error in the Funding statement. The funding statement for the article processing charge was displayed as:

“The author(s) declare financial support was received for the research, authorship, and/or publication of this article. The authors declare that this study received funding from Fresenius Medical Care. The funder was not involved in the study design, collection, analysis, interpretation of data, the writing of this article, or the decision to submit it for publication. The funding was received to pay the article processing charge.”

The correct Funding statement appears below.

